# Maternal-fetal emotional relationship during pregnancy, its related factors and outcomes in Iranian pregnant women: a panel study protocol

**DOI:** 10.1186/s12978-018-0620-6

**Published:** 2018-10-17

**Authors:** Vajihe Atashi, Shahnaz Kohan, Zahra Salehi, Kobra Salehi

**Affiliations:** 1 Adult Health Nursing Department, Faculty of Nursing and Midwifery, Isfahan University of Medical Sciences, Isfahan, Iran; 2 Nursing and Midwifery Care Research Center, Faculty of Nursing and Midwifery, Isfahan University of Medical Sciences, Isfahan, Iran; 3 Midwifery & Reproductive Health Department, Faculty of Nursing and Midwifery, Isfahan University of Medical Sciences, Isfahan, Iran

**Keywords:** Attachment, Maternal fetal attachment, Pregnancy, Factor, Outcomes, Longitudinal study

## Abstract

**Background:**

Considering the importance of attachment in child’s development and mother’s health, various related factors and also lack of necessary information in this regard in our country, the research team decides to conduct this study to evaluate maternal-fetal attachment during pregnancy, its changes and post-partum consequences on mother-infant relationship. This process should be studied during pregnancy and also after delivery so that the effect of related factors and the changes in attachment over time could be determined and comprehensible information about the effective underlying conditions on this issue would be gathered.

**Methods:**

The present research is a longitudinal study (panel study). Data gathering would start at the first trimester, continue during second and third trimesters of pregnancy, first visit after delivery, second, fourth and end sixth months later. Pregnant women in the first trimester would be selected and contacted. If they have inclusion criteria, they would be selected as a participant. At first, their demographic-reproductive characteristics, Beck Depression Inventory (BDI), Spielberger State-Trait Anxiety Inventory (SSTAI), The Social Support Appraisal (SSA), Adult Attachment Scale (AAS) and Parental Bonding Instrument (PBI) would be completed; during the second trimester, BDI, SSTAI and Cranley’s Maternal-Fetal Attachment Scale (CMFAS) would be completed. In the third trimester, the same questionnaires would be completed.

During the first month after birth, Avant’s questionnaire of Mother-Infant Attachment Behaviors would be completed. At the second, fourth and sixth months after delivery Muller’s Mother -Infant Attachment Scale would also be completed.

**Discussion:**

The results of the study will be provided to maternal child health policy-makers in the health system. This information could not be obtained through cross-sectional studies and through one episode of data collection and more studies are needed to provide us a perspective of the mother-infant relation over time. Studying attachment during pregnancy would provide us a chance to learn more about this process.

## Plain English summary

Attachment as a set of internal behaviors that would cause the infant to become close related to his/her main caregiver, who is usually the mother, was first introduced for the post-partum period, but it is believed that attachment starts long before birth, during pregnancy. According to the evidences, this relationship could be affected by different factors like nationality, cultural, mental and social conditions and individual’s past; namely women who have not experienced a secured attachment during their childhood might encounter problems in developing an attachment to their infant.

The review of literature about related factors to prenatal attachment among different groups of pregnant women have emphasized that despite the numerous evaluations and different methods of studies, further studies are required in this field.

Therefore considering the importance of attachment in child’s development and mother’s health and various factors that are effective on this matter and also lack of necessary information in this regard in IRAN, the research team decide to conduct this study to evaluate maternal-fetal attachment during pregnancy and its post-partum consequences on mother-infant relationship.

## Background

The attachment theory was first introduced by John Bowlby in 1960s about the mother-child bond [1]. He defined attachment as “a set of internal behaviors that would cause the infant to become closely related to his/her main caregiver, who is usually the mother” [2].

Although this concept was first introduced for the post-partum period, but it is believed that attachment starts long before birth, during pregnancy [3–5]. In other words, attachment starts when mother finds out about her pregnancy and this is the real start point for fetus’ dialog with his/her surrounding world [6]. Cranley has expressed mother’s attachment as her enthusiasm in behaviors for interacting with her fetus [7] and Muller believed that it is more than just a behavior and has defined as a unique relationship between the mother and her fetus [8]. The common characteristic between all of these definitions is emphasis on the importance of maternal-fetal relationship [9, 10]. Development of this relationship is important due to its role in creation of attachment after birth [11, 12]. Some researchers have reported that mother’s attachment to the fetus would develop during pregnancy and it would help her prepare for transition to the motherhood period [13, 14]. Some studies have shown that prenatal attachment is an important factor in predicting post-partum attachment behaviors [13], it is associated with mother-infant post-partum interactions and communications [11] and it has an important role in the health of the pregnant mother and her fetus [10]. Attachment, which is one of the essential needs of human beings, would act like an invisible connection and maintain a close relation between the mother and the child [15], is considered an important part of child’s development [1, 4, 16–23] and would develop a sense of trust in the child [5]. Results of studies conducted on human beings and other species have revealed that care deprivation has a major effect on the evolution of regulatory system and coping with stress. Mother’s reaction to child’s stress is considered an important source for coping; therefore infants with sensitive and respondent parents would learn that at the time of stress, parents are always available and it would be more chance for them to have secure attached relationship with their parents in the future. But infants with insensitive parents would learn that parents are not always available at the time of stress and crisis; so they would probably develop an insecure relationship with their parents [24].

Inappropriate development of attachment would have an adverse effect on the regulating part of child’s right brain; different related studies have reported lower emotional and mental development, weak social interactions, school refusal, and more aggressive and hostile behaviors during childhood, behavioral disorders during adolescence and more tendencies toward drug abuse during adulthood [10, 25].

Furthermore, mother’s attachment to her fetus has a close relation with other important processes. Meleis believed that being related and attached to the fetus and sacrificing for him/her is a responsibility that mothers should be committed to from the pregnancy period [26]. Mother’s commitment, attachment and preparation for taking care of their child during pregnancy are the main parts of the motherhood process and for reaching the maternal identity [26, 27].

Since the presentation of this concept in the early 1980s many studies have been conducted in this issue. Most of the studies have evaluated attachment in the form of mother-infant postpartum interactions [28–31], but a few has been dedicated to studying the subjective world of parents about their fetus during pregnancy [32] and mother’s attachment to her fetus is not well recognized [33]; so that most of the studies about attachment during pregnancy are cross-sectional and related to the third trimester [1, 10, 34–41]. Studying attachment during a limited time period would not be able to provide comprehensible information about its related factors and outcomes [39]; while, according to evidences, this relationship could be affected by different factors like nationality, cultural, mental and social conditions and individual’s past; namely women who have not experienced a secured attachment during their childhood might encounter problems in developing an attachment to their infant [12, 39]. Muller also believed that pregnant mother’s experience of attachment to her own mother during childhood, would affect her future attachments to the family, spouse and friends and this process is effective on accepting the pregnancy and attachment to the fetus [30]. In other words, the current living condition of people is rooted in their past and attachment as a unique process is affected by personal factors, individual’s beliefs and past and environmental and cultural factors; attachment could also be affected by different issues like mental condition, social support, mother’s age, gravid and etc. which vary among different cultures [41].

Since all of the mother’s behaviors, actions and thoughts during pregnancy could have more permanent effects on the fetus than any other period of child’s life [42] and also since pregnancy is considered a critical period in the development, therefore it is necessary to evaluate mother’s attachment to her fetus more accurately. Few quantitative and cross-sectional studies have been conducted in Iran about maternal-fetal attachment [41, 43–49]. In all of those studies mother’s attachment to her fetus has been studied at one time, so their attachment could not be evaluated comprehensibly in these studies.

Therefore considering the importance of attachment in child’s development and mother’s health and various factors that are effective on this matter and also lack of necessary information in this regard in our country, the research team decide to conduct this study to evaluate maternal-fetal attachment during pregnancy and its post-partum consequences on mother-infant relationship.

This process should be studied during pregnancy and also after delivery so that the effect of related factors and the changes in attachment over time could be determined and comprehensible information about the effective underlying conditions on this issue would be gathered. This study would reveal many facts about attachment and its related underlying and mediator conditions. Awareness about prenatal attachment is important in the health of pregnant mothers and their children and also in finding optimal compatibility with motherhood. The collected information not only would increase the perception of the medical team about the process of attachment, but it would also provide the possibility for planning proper interventions to improve maternal-fetal relationship.

## Methods

This process would be studied during pregnancy and also after delivery so that the effect of related factors and the changes in attachment over time could be determined and comprehensible information about the effective underlying conditions on this issue would be gathered. The present research is a longitudinal study (panel study). Data gathering would start at the first trimester, continue during second and third trimesters of pregnancy, first visit after delivery, second, fourth and end sixth months later.

### Objectives

The objectives are as follows:

1. Determining and comparing the mean score of MFA in the study subjects during second and third trimesters
2. Determining and comparing the mean score of state trait anxiety and depression in the study subjects at every trimester
3. Determining the mean score of social support in the domains of family, friends and others and parental bonding for all the subjects
4. Determining the frequency distribution of adults’ attachment style for all the subjects during first trimester
5. Determining and comparing the mean score of postpartum depression 4 and 8 weeks after birth
6. Determining the mean score of mother-newborn attachment during the first month after birth
7. Determining and comparing the mean score of mother-infant attachment at 2, 4 and 6 months after birth
8. Determining the relation between mother-fetal attachment and prenatal anxiety, depression, social support and parental bonding
9. Determining the relation between maternal-fetal attachment and mother-newborn attachment
10. Determining the relation between maternal-fetal attachment and mother-infant attachment
11. Determining the relation between maternal-fetal attachment and postpartum depression
12. Determining the demographic-reproductive characteristics of the subjects
13. Determining the relation between maternal-fetal attachment and demographic-reproductive characteristics

### Study design

#### The type of the study

The present research is a longitudinal study (panel study). Data gathering would start at the first trimester of pregnancy, continue during second and third trimesters of pregnancy, first visit after delivery, two, four and end 6 months later.

#### Study settings

This study would be conducted at selected health care centers of Isfahan, Iran.

#### Study participants

The participants of this study include all of the pregnant women under the maternity coverage of the selected health care centers who have the inclusion criteria.

#### Sample size

Based on the opinions of a statistician and considering the sample size of similar studies which were conducted in the world, also considering its applicability, the sample size was calculated to be 400.

#### The inclusion criteria

Iranian nationality, willing to participate, no history of severe mental disorders during the past year, no history of substance abuse and drug addiction, experiencing no severe stress during the past year (any serious diseases, death of a close relative, immigration).

#### The exclusion criteria

In case of unavailability for completing the information and pregnancy and postpartum follow-ups or the death of the mother or the fetus, the participant would be excluded from the study.

#### Sampling method

The sampling method for this study would be cluster randomized sampling. So that the province health centers 1 and 2 would each be considered as a cluster and then based on the number of pregnant women under their coverage, the required sample size for each cluster would be calculated. Then randomly some of health centers would be selected from each of the province health centers 1 and 2. Then at the selected health centers, purposive sampling would be used for inclusion of eligible subjects.

#### Data collection method

After taking the necessary permissions and introduction letters, the researchers would refer to the study settings and after providing complete explanations and taking permission from the health care centers’ authorities, the sampling process would be started. At each center, on the base of inclusion criteria, selective sampling would be conducted.

In every health care center, the researcher would first assess the electronic medical files of pregnant women. Pregnant women who are in the first trimester (gestational age < 13 weeks) would be selected and contacted. If they have inclusion criteria, they would be selected as a participant. Then the women would be invited to attending in the health care center. During the in-person visit, necessary explanations would be provided to the women and informed consent would be taken. Then their address, 2 cellphone numbers and a land line number were obtained. At first, their demographic-reproductive characteristics would be collected. Then Beck Depression Inventory (BDI) and Spielberger State-Trait Anxiety Inventory (SSTAI) would be given to them to complete. If mothers have enough time, the Social Support Appraisal (SSA), Adult Attachment Scale (AAS) and Parental Bonding Instrument (PBI) would also be given to them; if not, these questionnaires would be completed at their next visit for receiving routine prenatal care after coordinating with the researcher.

During the second trimester (14–26 weeks of pregnancy), according to the prior arrangement between the researcher and the women, BDI and SSTAI would again be completed by the woman during her visit-point in the health center. Also Cranley’s Maternal-Fetal Attachment Scale (CMFAS) would be completed. This questionnaire should be completed after mother has felt the movement of her fetus.

After coordinating with the woman, during the third trimester (27 weeks until the end of pregnancy), the same questionnaires as the second trimester would be completed.

During the first month after delivery, after coordinating with the mother through phone calls, Avant’s questionnaire of Mother-Infant Attachment Behaviors would be completed during the mother and infant’s first visit to the health care center. The same way, during the 2nd, 4th and 6th months after delivery Muller’s Mother -Infant Attachment Scale would also be completed. Edinburgh Postnatal Depression Scale would be completed 4 and 8 weeks after delivery. Women would be monitored regarding their relation with their infant for 6 months after delivery. This period was selected since during the first 6 months, creation and development of attachment would be completed. During this period, the infant would be able to give different social responds to different people. The infant would be able to recognize familiar individuals and be more responsive to them than others. Mainly, the infant is focused on her/his main caregivers. The mother and the infant present mutual reactions to each other and get more attached in this process; infant’s mood would be regulated and maintained by mother’s reactions (16).

It must be noted that to provide same conditions, the deadline for selecting and enrolling the participants at each health care center was set at 3 months.

## Data management and statistical analysis

### Data collection instruments

The required information for this study would be collected through questioning and completing the mentioned questionnaires.

In this study data collection instruments are demographic-reproductive characteristics questionnaire, Cranley’s Maternal-Fetal Attachment Scale, Vaux Social Support questionnaire, Collins and Read Adult Attachment Scale, Beck Depression Inventory, Edinburgh Postnatal Depression Scale, State-Trait Anxiety Inventory by Spielberger, Parental Bonding Instrument: mother’s form, Avant’s Observational Checklist for mother-newborn interactions and Muller’s Mother Attachment Inventory.

- Demographic-reproductive characteristics of the participants would be gathered through the questionnaire. This questionnaire has three parts: demographic characteristics, obstetric and delivery characteristics.
- Cranley’s Maternal-Fetal Attachment Scale: This questionnaire has 24 items and mother’s feelings toward each phrase would be scored from 1 to 5 (definitely yes: 5, yes: 4, not sure: 3, no: 2 and definitely no: 1). Item 22 is scored in reverse. The lowest score for the questionnaire is 24 and its highest score is 120. Its validity has been approved through content validity by Khoramroudi and its reliability has been approved by test-retest method with a correlation coefficient of 0.85 [46].
- The Social Support Appraisal by Vaux et al. has 23 items which includes three domains of family, friends and acquaintances. Family and friends subscales each contain 8 items and acquaintances subscale contains 7 items. Reliability coefficients of this questionnaire were 0.7 and 0.9 for two different groups of students [50].
- Adult Attachment Scale by Collins and Read has 18 questions with Likert scale. The answers vary from totally disagreed (definitely No) to totally agreed (completely match my characteristics). Each item would be answered with a 5-point likert scale. Items 5, 6, 8, 16, 17 and 18 are answered in reverse. Validity and reliability of this scale have been approved by Pakdaman [51] with a Cronbach’s α of 95%.
- Beck Depression Inventory: This questionnaire contains twenty-one items multiple choice questions and each could be scored from 0 to 3. Mothers who would gain a score of 21 or more would be considered depressed. This questionnaire has been validated in Iran by Ghasem Zadeh and approved as a valid tool for evaluation of depression [52].
- Edinburgh Postnatal Depression Scale: This questionnaire has been used in Iran in 1993 by Namazi with a reliability of 90% [53]. Also validity and reliability of this questionnaire was determined to be 0.75 in 1997 by Khodadoostan in Isfahan University [50]. This inventory contains ten items 4-choice questions. The choices for each question are scored from 0 to 3. The total score would be achieved by summing all the scores and would range from 0 to 30. Questions 1, 2, and 4 are scored from 0 to 3 and the rest from 3 to 0. Gaining a score of 12 or more would be considered as having postpartum depression [53].
- The Spielberger State-Trait Anxiety Inventory (STAI): This scale contains 20 phrases and the individual should answer each within a 4-ponit Likert scale (almost never, sometimes, most of the times and almost always) with their general feelings about the phrase. Score of 20 to 31 indicates mild anxiety, score of 32 to 42 indicates lower moderate anxiety, 43 to 52 indicates higher moderate anxiety, 53 to 62 indicates relatively severe anxiety, 63 to 72 indicates severe anxiety and a score 73 to 80 indicates extremely severe anxiety. The Cronbach’s α for this scale is 0.90. The reliability of State-Trait Anxiety Inventory has been calculated to be 0.73 to 0.86 through test-retest method for 1 h to 104 days. Panahi-Shahri reported a correlation coefficient of 0.84 for this scale through test-retest method [54].
- Parental Bonding Instrument (Mother’s form): This questionnaire was first developed by Gordon Parker, Hillary Tupling and L.B. Brown. It has 25 items and 2 subscales of caring (12 questions) and excessive support (13 questions), which is used for evaluating family bonding. This scoring method is based on 4-point Likert scale and the scores of 0, 1, 2 and 3 are assigned to the choices of “very much”, “almost much”, “almost little” and “very little” respectively. Higher scores mean that the parents are more attendant and lower scores indicate cold rejecting parents. Questions 1, 5, 6, 8, 9, 10, 11, 12, 13, 17, 19, 20 and 23 are scored in reverse. This questionnaire has a well-approved validity and reliability in Iran [55].
- Muller’s Mother Attachment Inventory: This scale has 26 items. Its validity has been approved through content validity and its reliability has been approved with a Cronbach’s α of 0.89 [16].
- Avant’s questionnaire of Mother-Infant Attachment Behaviors: This questionnaire included three groups of behaviors: emotional behaviors, proximity behaviors, and caring behaviors, observed for 15 min. In the first 30 s of each minute, the behaviors were observed and during the second half, these behaviors were recorded; each observed behavior was recorded once per minute. In total, the observation took 15 min; therefore each behavior was observed for a maximum of 15 times in 15 min. In total, 11 behaviors could be observed in 15 min and each observed behavior in one minute was allocated one score (total score = 165). The sum of scores presented mother-infant attachment and higher scores indicated stronger attachment. This scale was first developed by Avant and its reliability has been reported to be 0.98 in Iran [56].

### Data analysis

The collected data will be analyzed using descriptive statistical methods (mean, standard deviation and frequency) and inferential statistics (independent t, paired t-test, Chi-square, Pearson’s correlation coefficient, repeated measurement (MANOVA), Fisher’s exact test, Wilcoxon and Mann-Whitney) and by using SPSS 16 software.

## Discussion

Based on the results, it is possible to provide appropriate strategies for enhancing maternal-fetal attachment and improve the provision of care in the health of pregnant women and their infants in the health care system. The review of literature about related factors to prenatal attachment among different groups of pregnant women have emphasized that despite the numerous evaluations and different methods of studies, further studies are required in this field [57]. For example social support is one of the issues that its effect has not been conclusively determined [25]. Also longitudinal studies are required to evaluate the effect of attachment during pregnancy on mothers’ mental health, parental behaviors and the consequences of infancy. This information could not be obtained through cross-sectional studies and through one episode of data collection [30] and more studies are needed to provide us a perspective of the mother-infant relation over time [58].

Walsh has stated that predictive factors for mother-fetal relationship have yet not been determined conclusively [59]. By determining mothers’ attachment style and the status of maternal-fetal attachment during pregnancy, in time interventions and educations could be planned for improving these interactions and consequently improve the mother-child attachment during the sensitive period of growth [60]. The prenatal period is a proper chance for evaluating maternal-fetal attachment. Studying attachment during pregnancy would provide us a chance to learn more about this process. According to Bowlby studying this issue would help us understand and cope with the psychological problems during and after pregnancy [10].
